# Understanding alveolarization to induce lung regeneration

**DOI:** 10.1186/s12931-018-0837-5

**Published:** 2018-08-06

**Authors:** José Alberto Rodríguez-Castillo, David Bravo Pérez, Aglaia Ntokou, Werner Seeger, Rory E. Morty, Katrin Ahlbrecht

**Affiliations:** 1 Member of the German Lung Research Center (DZL), Department of Lung Development and Remodelling, Max Planck Institute for Heart and Lung Research, Parkstrasse 1, 61231 Bad Nauheim, Germany; 2 Member of the German Lung Research Center (DZL), Department of Internal Medicine (Pulmonology), University of Giessen and Marburg Lung Center (UGMLC), Klinistrasse 33, 35392 Giessen, Germany

**Keywords:** Alveolarization, Neo-alveolarization, Regeneration

## Abstract

**Background:**

Gas exchange represents the key physiological function of the lung, and is dependent upon proper formation of the delicate alveolar structure. Malformation or destruction of the alveolar gas-exchange regions are key histopathological hallmarks of diseases such as bronchopulmonary dysplasia (BPD), chronic obstructive pulmonary disease (COPD), and pulmonary fibrosis; all of which are characterized by perturbations to the alveolo-capillary barrier structure. Impaired gas-exchange is the primary initial consequence of these perturbations, resulting in severe clinical symptoms, reduced quality of life, and death. The pronounced morbidity and mortality associated with malformation or destruction of alveoli underscores a pressing need for new therapeutic concepts. The re-induction of alveolarization in diseased lungs is a new and exciting concept in a regenerative medicine approach to manage pulmonary diseases that are characterized by an absence of alveoli.

**Main text:**

Mechanisms of alveolarization first need to be understood, to identify pathways and mediators that may be exploited to drive the induction of alveolarization in the diseased lung. With this in mind, a variety of candidate cell-types, pathways, and molecular mediators have recently been identified. Using lineage tracing approaches and lung injury models, new progenitor cells for epithelial and mesenchymal cell types – as well as cell lineages which are able to acquire stem cell properties – have been discovered. However, the underlying mechanisms that orchestrate the complex process of lung alveolar septation remain largely unknown.

**Conclusion:**

While important progress has been made, further characterization of the contributing cell-types, the cell type-specific molecular signatures, and the time-dependent chemical and mechanical processes in the developing, adult and diseased lung is needed in order to implement a regenerative therapeutic approach for pulmonary diseases.

## Background

Therapeutic options for diseases that cause perturbations to the lung structure such as bronchopulmonary dysplasia (BPD), chronic obstructive pulmonary disease (COPD), and pulmonary fibrosis, are limited; and as such, new therapeutic concepts are needed [1–4]. A translational regenerative approach represents one future promising option for the development of new therapeutic concepts. In this approach, the identification of key molecular and cellular drivers of alveolarization and neo-alveolarization would be used to induce regeneration of alveoli in the diseased lung. The aim of this review is to provide an overview of recent developments in the underlying concepts of alveolarization and neo-alveolarization, and to explain how this knowledge might be used to induce regeneration of alveoli. Furthermore, techniques currently available to approach this question are highlighted. Current knowledge of alveolarization and neo-alveolarization includes consideration of the contributing cell-types, extracellular matrix (ECM) components and selected molecular mediators [5–14]. However, studies that have assessed cell-lineage specification, progenitor- or stem-cell characteristics, and molecular signatures in relation to the localization of a cell are limited. Future directions for research supporting this regenerative approach remain a crucial topic to be discussed, and likely directions are highlighted in the last paragraph of this review.

## Main text

### Alveolarization

Alveolarization represents a process during lung development that leads to the formation and maturation of the distal parts of the lung: the alveoli. In rats and mice, alveolarization takes place postnatally, whereas in humans, alveolar development begins prior to birth [3, 15–20]. Due to the limited availability of human tissue, most of the studies dissecting the principles of alveolarization have been conducted in rodents. At birth, the murine lung is in the saccular stage, which lasts from embryonic day (E)18.5 until postnatal day (P)5 [5], and which is comparable to the stage of lung development in which most pre-term born human infants are undergoing at the time of premature rupture of membranes. As such, term-born mouse and rats are often used to model the lung in pre-term born infants, with the important caveat that these lungs are perfectly competent for effective gas exchange at birth, contrasting with the situation in pre-term born human infants.

### Nature of alveolar epithelial cells during the saccular stage and alveolarization

**Fig. 1:**
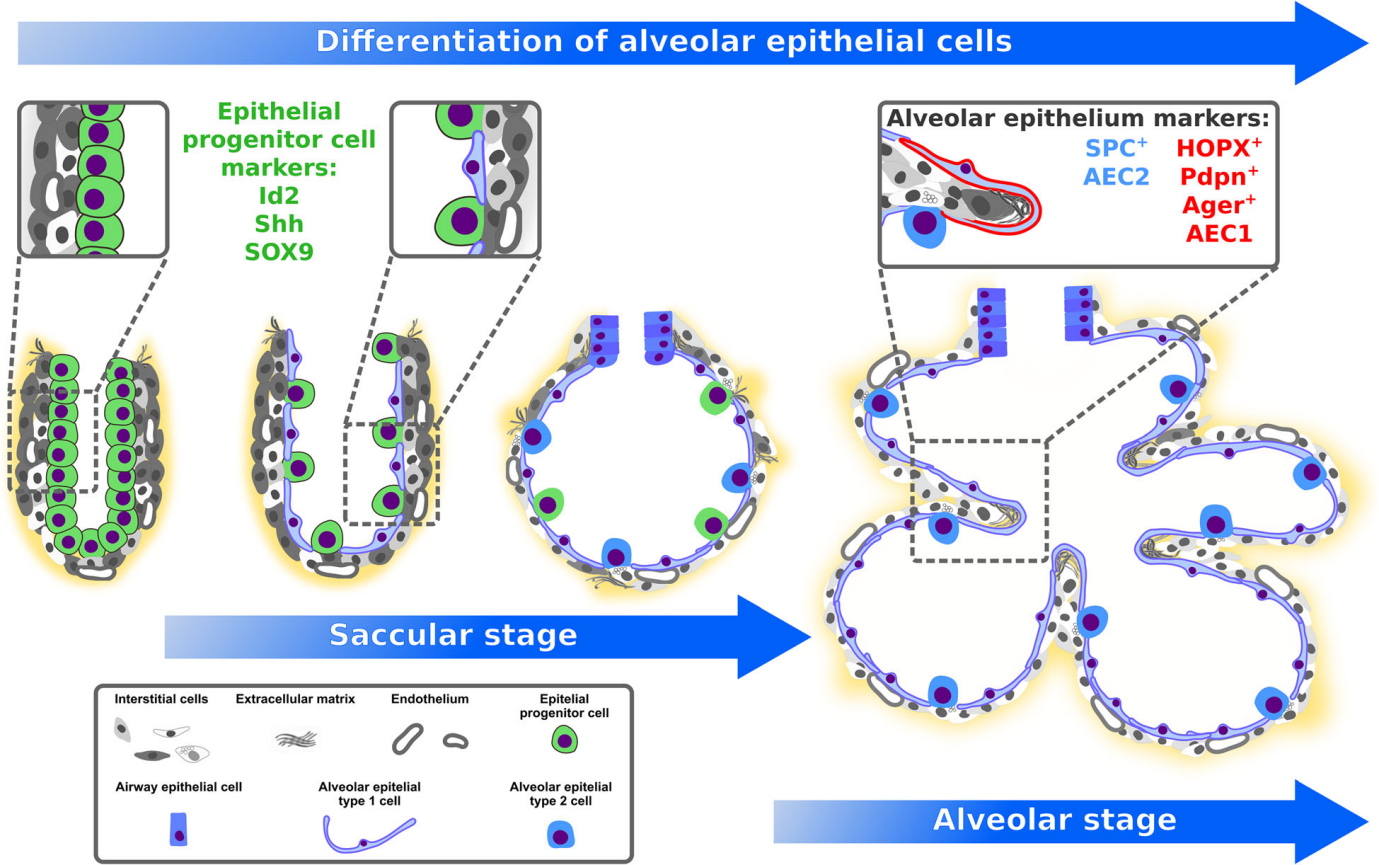
Alveolar epithelial cells during alveolarization. During the saccular stage, alveolar epithelial type I cells (AECI) and alveolar epithelial type II cells (AECII) are derived from a common bipotent progenitor cell. After differentiation single AECl can cover multiple alveoli during alveolarization and in the adult lung

During the saccular stage of lung development, distal parts of the lung contain air sacs (sacculi or saccules) which are lined with an epithelial layer that originates from the foregut endoderm, and consists of differentiated alveolar epithelial type I cells (AECI) and alveolar epithelial type II cells (AECII) [5]. During the saccular stage, AECl and AECII are derived from a common bipotent progenitor cell (Fig. 1) [21]. As described in a later section of this review, mechanical forces and fibroblast growth factor (FGF) 10 are amongst recently-identified regulators of this differentiation process [22–24]. After alveolarization, and in the adult lung, AECII acquire stem cell properties and are capable of self-renewal to replace AECl after injury [21, 25]. Single-cell sequencing of distal lung epithelial cells during the saccular stage confirmed a bipotent progenitor for AECl and AECll and revealed further cell-type specific markers and subpopulations during the process of differentiation [26]. Cuboidal AECII cells are capable of producing surfactant proteins and lipids which decrease the surface tension of the alveoli [5, 27]. The cell surface of squamous AECl expands drastically during the alveolar stage [28]. One AECl covers multiple alveoli during alveolarization and in the adult lung (Fig. 1) [28–30]. Due to the close proximity of AECI to the capillary network, and the comparatively larger surface area when compared to AECII, AECl represent the site of gas exchange [30].

### Role of mesenchymal cells and extracellular matrix during alveolarization

**Fig. 2:**
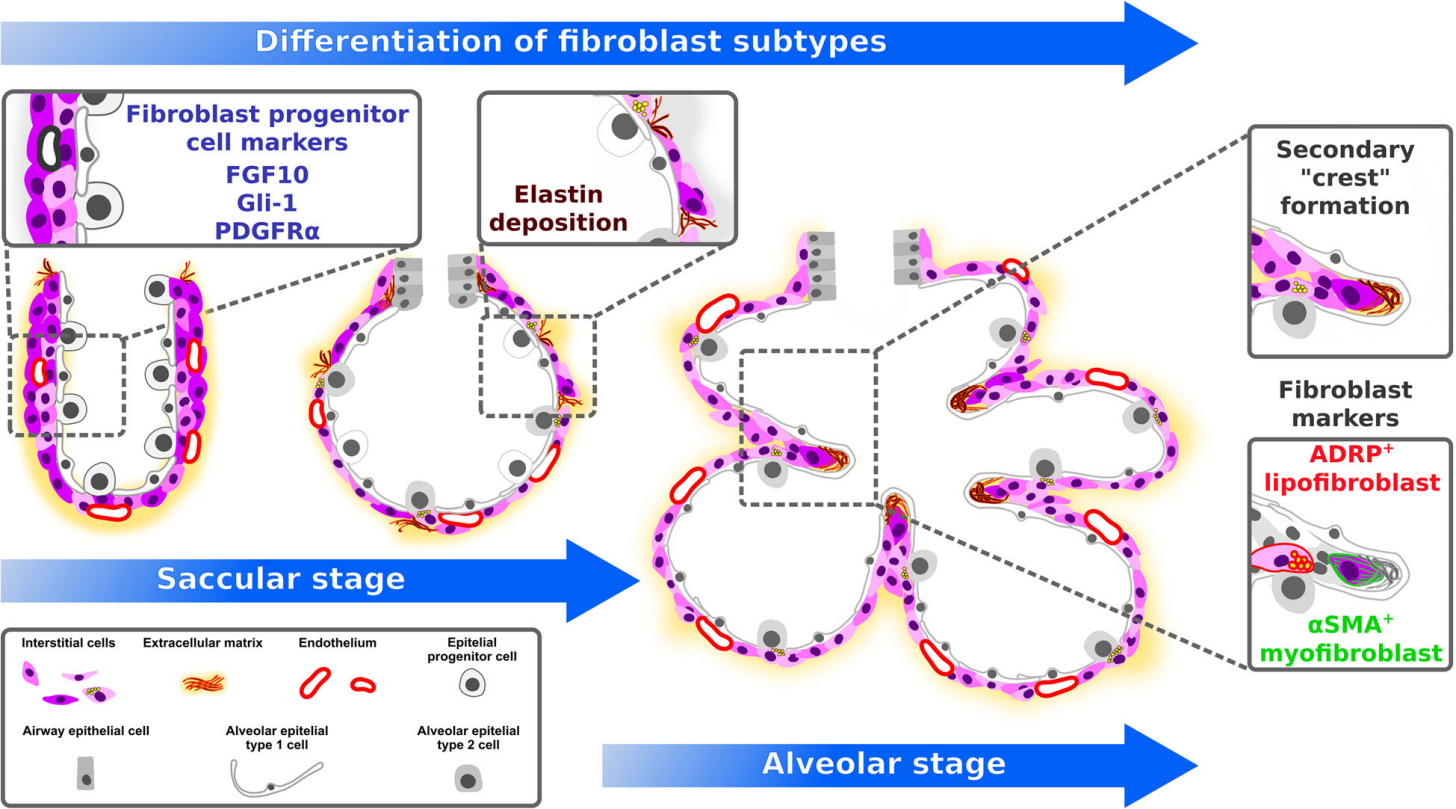
Mesenchymal cell-types during alveolarization. Alveolar mesenchymal fibroblasts such as myofibroblasts and lipofibroblasts differentiate from mesenchymal progenitor cells. Key cell lineages involved are the platelet-derived growth factor receptor (PDGFR)α lineage, the fibroblast growth factor (FGF)10 lineage and the GLI-Kruppel family member (Gli-1) lineage. Elastin deposition by myofibroblasts takes place at the so called “secondary crests” visualized in 2D lung sections

Further components of the alveolar air-sac walls (primary septa) are endothelial cells that originate from the lung endoderm [8, 31, 32], and a variety of interstitial cell-types such as fibroblasts, which originate from the lung mesoderm [8, 33–35]. In rat lung fibroblasts, an increase in retinoic acid levels has been demonstrated during secondary septation [36], and retinoic acid has been proposed to impact elastin production [36]. These studies highlighted a potential regenerative activity of retinoids in the lung. Retinoic acid administration to rats has been demonstrated to promote postnatal alveolarization, and to attenuate elastase-induced pulmonary emphysema [37, 38]. During the saccular stage, elastin expression increases, and elastin deposition by fibroblasts takes place [39]. In rodents, by P4, so-called secondary septa appear in the primary septa at sites of elastin deposition [5, 31]. At the tip (secondary crest) of these still-immature secondary septa, α-smooth muscle actin (αSMA)^+^ myofibroblasts appear, and the expression of ECM components such as elastin further increases (Fig. 2) [5, 6, 33, 39]. One recent study has carefully dissected structural changes in the developing alveoli, and elastin localization during alveolarization, using 3D imaging techniques [14]. These analyses pointed out that secondary crests arise as ridges into the alveolar air-sac lumen. An organized network resembling a “fishnet” composed of αSMA and the ECM component elastin runs within the ridges [14]. Quite similar observations were made in a further study that analyzed the spatial and temporal changes in elastin and laminin distribution during alveolarization [40]. That study demonstrated that elastin fibers formed ring-like structures which were localized to the saccular openings, and later on were interconnected by further elastin fibers [40]. Crosslinking of ECM components has been demonstrated to be altered during aberrant lung development [41]. The downstream signaling molecule of the sonic hedgehog pathway, Gli-1, has been demonstrated to label a cell-lineage which gives rise to secondary crest myofibroblasts (Fig. 2) [42–44]. The deposition of ECM components and the presence of alveolar myofibroblasts seems to be an attribute for secondary septation and has been demonstrated to be dependent on platelet-derived growth factor (PDGF)-A signaling [6, 45]. The ligand PDGF-A is produced by epithelial cells and signals via the cognate receptor PDGF receptor (PDGFR)α, which is expressed by mesenchymal cells [6, 46, 47]. In vivo labeling and lineage-tracing studies of PDGFRα^+^ cells have demonstrated that pulmonary PDGFRα^+^ cells serve as progenitor cells for peribronchial and alveolar myofibroblasts, as well as for a proportion of the pulmonary lipofibroblasts, and are present in the primary and secondary septa (Fig. 2) [6, 45, 48, 49]. Further studies have highlighted the progenitor nature of PDGFRα^+^ cells for peribronchial smooth muscle cells, myofibroblasts and lipofibroblasts during prenatal lung development and alveolarization using time-series RNA-Seq analyses and immunophenotyping [50, 51]. Another recent study has described the spatiotemporal distribution of PDGFRα and the two PDGF ligands PDGF-A and PDGF-C over the course of lung development [52]. Expression of the ligands was detected in epithelial and smooth muscle cells, whereas the expression of PDGFRα was located to different mesenchymal cell populations [52], which is consistent with the prevailing view that epithelial-mesenchymal interactions are key mediators of lung development.

### Possible role for lipogenic versus myogenic fibroblast phenotypes during alveolarization

**Fig. 3:**
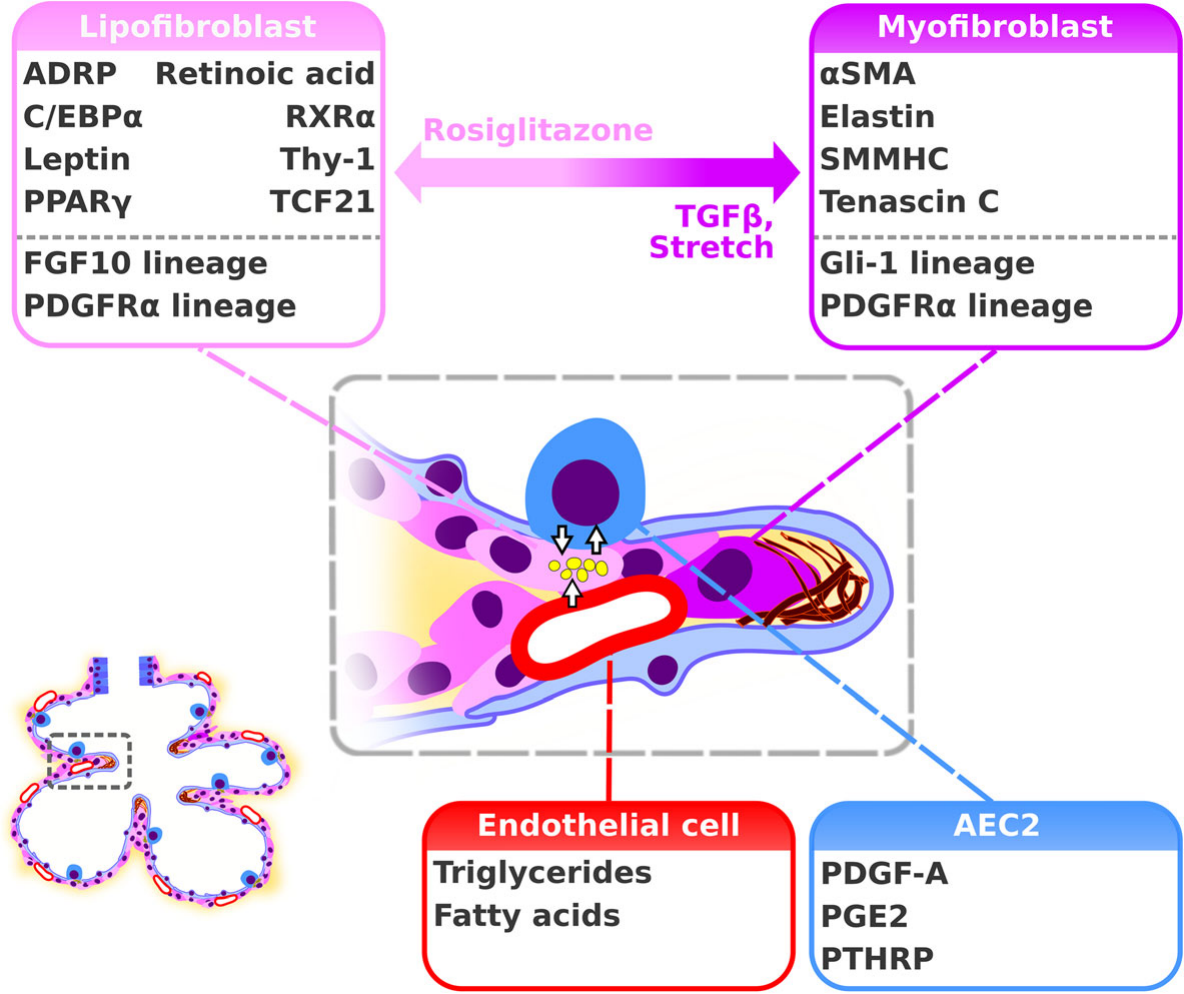
Lipogenic versus myogenic fibroblast phenotype. Lipogenic (lipofibroblast) and myogenic (myofibroblast) fibroblasts differentiate during early lung development and the saccular stage. Lipofibrobasts support alveolar epithelial type II cells (AECII) cell function via an intercellular crosstalk mediated by stretch, parathyroid hormone-related peptide (PTHRP), prostaglandin E2 (PGE2) and leptin while myofibroblasts produce extracellular matrix molecules such as elastin. Activation of peroxisome proliferator activated receptor (PPAR)γ by Rosiglitazone promotes the lipogenic phenotype. Stretch and transforming growth factor (TGF)-β induce the myogenic phenotype

Lipofibroblasts are also located within the primary and secondary septa in close proximity to AECII [48, 49, 53]. Lipid droplets and the expression of adipocyte differentiation related protein (ADRP, encoded by the *Plin2* gene) represent phenotypic characteristics of lipofibroblasts [53–57]. Further molecules such as peroxisome proliferator-activated receptor (PPAR)γ, cellular retinoic acid binding protein (CRABP), and the transcription factor TCF21 are expressed by lung mesenchymal lipofibroblasts (Fig. 3) [36, 58–61]. Lineage-tracing of FGF10^+^ cells revealed labeling of a subset of the lipofibroblast population [62]. Furthermore, lipofibroblasts have been demonstrated to support the synthesis of surfactant in AECII by providing triacylglycerols to AECII in a leptin- and stretch-dependent manner (Fig. 3) [53, 63]. During the period of secondary septation, the number of lipofibroblasts has been demonstrated to increase and peak at the same time as the peak of secondary septation, at P7 [64]. Activation of PPARγ using rosiglitazone (which promotes the lipofibroblast phenotype) in rat pre- and post-natally has been demonstrated to be protective against the structural changes that occur during the development of hyperoxia-induced lung injury [65, 66]. However, the presence of lipofibroblasts in the human lung remains controversial [54, 57, 67]. In contrast to rosiglitazone, which induces a lipogenic phenotype; Other reagents, stimuli and factors such as nicotine, mechanical forces, PDGFRα and transforming growth factor (TGF)-β have been demonstrated to induce a myogenic phenotype: nicotine treatment of isolated fibroblasts in vitro led to a myogenic phenotype (differentiation from lipofibroblasts to myofibroblasts) and could be reversed by rosiglitazone treatment [68]. Furthermore it has been demonstrated that mechanical forces could stimulate the differentiation of fibroblasts towards myofibroblasts (Fig. 3) [69]. Expression and activation of PDGFRα has also been demonstrated be involved in driving fibroblast differentiation towards a myogenic phenotype [6, 49, 70]. The expression of the PDGFRα chain has been demonstrated to be regulated by TGF-β (Fig. 3) [71]. A very recent study has also carefully dissected the role of the ligand PDGF-A using mice carrying a floxed *Pdgfrα* allele in combination with a *Sftpc*-Cre mouse strain [72]. The authors demonstrated that PDGF-A was needed for myofibroblast formation and proliferation as well as for the regulation of AECII proliferation [72]. Taken together, tightly regulated differentiation of alveolar fibroblasts towards the myogenic or the lipogenic phenotype seems to be relevant for secondary septation. Furthermore, neuropillin 1 has been demonstrated to impact the PDGF-A axis via activation of Src kinases and to be required for alveolar mesenchymal cell migration [73]. Following this line, a reduced expression of PDGFRα and PDGFRβ has been demonstrated in mesenchymal cells of infants who develop BPD [74]. Furthermore, the signaling via FGF members has been demonstrated to impact the formation of myofibroblasts from PDGFRα^+^ cells, as well as alveolar regeneration per se [75]. Deficiency of FGF10 has been demonstrated to be causative for the lethality in a mouse model of BPD [76]. Another mediator, Thy-1, which is expressed on lymphocytes and fibroblasts, has been demonstrated to severely impact alveolarization: an arrest of alveolarization itself has been demonstrated upon global loss of Thy-1 (CD90) [7]. The glycoprotein Thy-1 inhibits TGF-β activation, which leads to a reduced myogenic phenotype [7]. Since Thy-1 also is expressed on lipofibroblasts it might represent a molecule impacting on the balanced appearance of lipogenic and myogenic fibroblasts during secondary septation (Fig. 3) [7, 48]. However, the role of leucocytes might be of relevance since inflammatory cells have been proposed to play a role – and to be present – during lung development [77], and resident alveolar macrophages have recently been implicated as master regulators of arrested lung development [78]. Apart from the role of lipogenic and myogenic phenotypes of fibroblasts in lung development, lipogenic and myogenic fibroblast phenotypes are also involved in the progression and resolution of pulmonary fibrosis, which has been demonstrated in a murine model of bleomycin-induced lung fibrosis [79]. This mechanism supports the hypothesis that understanding the nature and development of lipogenic versus myogenic fibroblast phenotypic transformation might help to develop new therapeutic strategies for pulmonary diseases.

### Epithelial-mesenchymal interactions during alveolarization

The Interaction between mesenchymal and epithelial cells has been demonstrated to be essential for cellular differentiation and function during alveolarization. The formation of alveolospheres by alveolar epithelial cells (AEC) has been demonstrated to be supported by PDGFRα^+^ cells in vitro [25]. In line with these findings, a mesenchymal cell population from the Axin2 cell-lineage which expressed PDGFRα has been demonstrated to support AECI and AECII differentiation and function [12]. In contrast, a rare population of AECII which express Axin2 has been demonstrated to have alveolar stem cell activity in the adult lung and to be supported by juxtacrine Wnt signals from neighboring fibroblasts during homeostasis, and autocrine Wnt signals upon severe injury [80]. Further evidence for mesenchymal-epithelial interactions being key drivers of cell differentiation during alveolar development has emerged from analyses comparing two distinct mesenchymal cell populations of the leucine-rich repeat-containing G protein-coupled receptor (LGR)5 and LGR6 cell lineage: the LGR5 and LGR6 lineages have been demonstrated to support cell function and differentiation of either alveolar or bronchiolar epithelial cells [13]. Furthermore human alveolar fibroblasts have been demonstrated to exhibit direct intercellular contact with AECl, AECll, capillaries and pericytes [81]. This strategic localization might position fibroblasts to mediate crosstalk between the epithelial and the capillary endothelial cells, as well as epithelial-mesenchymal interactions which are key drivers of cellular differentiation and function during alveolarization.

### Significance of mechanical forces in alveolarization

Further key drivers of cellular processes that promote alveolarization are mechanical forces. An understanding of lung alveolarization is based primarily on understanding processes such as cellular differentiation, localization, production of ECM molecules and underlying signaling cascades. However, the impact of mechanical forces on these processes has been analyzed in the context of intercellular crosstalk and the differentiation of alveolar epithelial cells [22–24, 63, 82]. Surfactant production of AECII has been demonstrated to be stretch-dependent via a stretch-induced de novo synthesis of phosphatidyl choline by AECII [63]. Furthermore, stretch-dependent interactions with fibroblasts via parathyroid hormone-related peptide (PTHRP), prostaglandin E2 (PGE2) and fibroblast-derived leptin increased surfactant synthesis (Fig. 3) [63, 82]. A very recent study carefully dissected the role of mechanical forces on alveolar epithelial cell differentiation using live imaging techniques [23]. Mechanical forces generated by inhalation of amniotic fluid by prenatal breathing movements were essential for the differentiation of AECI [23]. Furthermore FGF10/FGFR2 signaling has been demonstrated to prevent flattening of alveolar progenitor cells, protecting AECII differentiation [23]. Mechanical forces have also been demonstrated to impact airway tube morphogenesis by controlling cell shape and orientation of cell division of the airway epithelium [22]. Taken together, mechanical forces induced by pre- and post-natal breathing movements are essential for cellular differentiation processes of epithelial and mesenchymal cells during alveolarization.

### Different types of alveolarization and maturation of the secondary septum

Analyses of the temporal dynamics of the lung transcriptome during lung development revealed that changes in the transcriptome profiles confirmed previously defined stages of lung development [83]. Additionally, four stages of postnatal alveolar development were suggested based on the temporal changes in the lung transcriptome [83]. Similarities and contrasts of developmental processes and processes regulating disease and tissue homeostasis in the adult lung await to be dissected. For example, in contrast to the “classical or bulk alveolarization” which is initiated from an immature primary septum, “continued alveolarization” has been demonstrated starting from a more mature septum from P14 [31]. Maturation of the immature secondary septa by thinning of the septa and remodeling of double capillary networks to a single capillary network occurs during the stage of microvascular maturation starting at P12 [31]. During the stage of microvascular maturation, the laminin network has been demonstrated to be simplified, to ensure septal thinning [40]. During the last period of septal maturation by P21 mature septa appear next to continued alveolarization until young adulthood [31]. Taken together, understanding processes driving different types of alveolarization and the maturation of the alveolar septum might help to identify cellular and molecular drivers for each period, which might be used to induce regeneration of the alveolus in the diseased lung in which either regrowth or thinning of the alveolar wall is required.

### Conclusion alveolarization

Different cell-types contributing to secondary septation have been identified, but distinct cell-type specific functions against the background of chemical and mechanical conditions during lung development still need to be understood. Identification of progenitor cells and a detailed characterization of the differentiation and function of mesenchymal lineages are needed to better understand secondary septation. Furthermore, understanding the function of AECll and AECl during secondary septation, and mapping the molecular signatures of the interactions between alveolar mesenchymal and epithelial cells might provide further insights into the nature of alveolar septation.

### Neo alveolarization

The formation of new alveoli in the adult lung has been demonstrated in a variety of species including humans, after removal of a part of the lung (by pneumonectomy, PNX) [84–87]. In mice, left-sided PNX leads to a complete restoration of the mass-specific lung volume and total alveolar surface area within 21 days after the operation [86]. In humans, there is evidence for compensatory lung growth after PNX, but the time-course is months to years and a complete restoration of the lung capacity has not been demonstrated [84]. Some underlying mechanisms contributing to compensatory lung growth have been demonstrated in rodents, such as mediators of the alveolar stem cell niche and of vascular- epithelial interactions and will be discussed in the following paragraphs.

### Nature of alveolar epithelial cells during neo-alveolarization

In adult mice it has been demonstrated that HOPX1 lineage-labeled AECl expressed surfactant protein C (SPC) 21 days after PNX suggesting the generation of AECll from AECl during neo-alveolarization after PNX [10]. Furthermore a SPC^−^AEC progenitor cell pool has been identified in an in vivo embryonic lung organoid assay in mice suggesting a further progenitor cell-lineage for AECII [88]. However there is strong evidence based on lineage tracing approaches that AECll hold stem cell features in the adult and postnatal lung [21, 25]. A further very recent study carefully dissected the role of bone morphogenic protein (BMP) signaling during alveolar regeneration in organoid culture and in vivo during the PNX model [89]. In the alveolar stem cell niche which, consists of AECII and PDGFRα-expressing fibroblasts [90], BMP signaling was demonstrated to regulate AECII support function of PDGFRα^+^ fibroblasts and differentiation of AECI and AECII, as well as AECII proliferation and self-renewal [89].

### Role of the vascular system and vascular mediators during neo-alveolarization

Similar to the bulk alveolarization that occurs during postnatal lung development, crest formation arising from preexisting septa involving capillaries and mesenchymal cells has been demonstrated using scanning electron microscopy of vascular casts after PNX [91]. Mechanisms driving neo-vascularization such as sprouting and intussusceptive angiogenesis resemble neo-vascularization during postnatal bulk alveolarization [91]. Platelet-derived stromal-cell-derived factor (SDF) has been demonstrated to impact AEC expansion and neo-alveolarization after PNX [9]. Likewise, the crosstalk between pulmonary capillary endothelial cells and AEC during neo-alveolarization after PNX has been demonstrated to involve vascular endothelial growth factor receptor (VEGFR)2, matrix metalloproteinase (MMP)-14 and epidermal growth factor receptor (EGFR) resulting in expansion of epithelial progenitor cells [92]. Taken together, growth of the vascular system, and vascular mediators and growth factors such as SDF-1 and VEGF-A, are further drivers of epithelial cell function during lung regrowth after PNX.

### Impact of mesenchymal cells, mechanical forces and innate lymphoid cells on neo-alveolarization

Further key drivers of lung growth after PNX have been identified in mesenchymal lineages. There is evidence for the participation of PDGFRα^+^ cells in compensatory lung regrowth [93, 94]. Gene expression profiling of postnatal and adult mouse lungs undergoing PNX revealed concordantly as well as variably regulated genes [95]. There is a growing body of evidence that features of bulk alveolarization occur during compensatory lung growth of the adult lung, but alternative mechanisms need to be considered in addition. Cell proliferation, change in mechanical forces, ECM remodeling and the activation of different signaling pathways have been demonstrated in response to PNX [86, 92, 95–97]. Finally, myeloid cells and type 2 innate lymphoid cells (ICL2) have been demonstrated to hold regenerative capacity during compensatory lung growth after PNX [11]. A variety of cellular and molecular factors impacting on neo-alveolarization after PNX has been identified. However, target molecules capable to initiate neo-alveolarization have not been recognized. Further combined lineage tracing, lineage ablation and cell type specific loss or gain of function studies are needed to identify hierarchies and functions of cell types and signaling cascades driving neo-alveolarization in the adult lung.

### Models to approach lung regeneration

The induction of neo-alveolarization in the diseased lung requires a detailed knowledge of the conditions which are necessary for alveolar septum formation. Therefore, the identification of suitable models to study alveolar septum formation is essential. Transgenic tools [98–100] and genome-wide screening approaches [83] have revealed a variety of contributing cellular and molecular candidates and thus represent suitable tools to elucidate relevant candidate drivers for alveolarization. Therefore, alveolariaztion and neo-alveolarization after PNX are currently analyzed primarily in mice in vivo*.* However, some disadvantages of both models remain, and these are discussed in the following sections.

### Analyzing alveolarization

Mice represent a valuable tool to analyze the process of alveolarization since alveolarization largely takes place postnatally in mice. Advantages of analyzing alveolarization in vivo in mice are the possibility to perform cell-type specific and inducible lineage tracing, cell and gene modulation, as well as cell ablation based on the CreER^T2^/*loxP* system [101]. Differentiation processes, stem cell features, and morphological appearance can be analyzed in combination with high quality imaging approaches. Even cell-lineage and single-cell sequencing analyses can be performed at different time-points of alveolarization. However, a key limitation of studying alveolarization in mice is the need to validate identified candidate cells and genes in human tissue, which has to be performed to ensure the ranslational value of the pathway identified. Furthermore, it remains to be discovered which candidates relevant for alveolarization in postnatal mice might also be relevant for lung regeneration. Taken together, analyzing alveolarization in mice in vivo represents a suitable model, but validation of candidate factors in human lung tissue and adult mouse lung tissue has to be considered.

### Elucidating neo-alveolarization using the PNX model

Exploitation of the model of compensatory lung growth after PNX will provide a better understanding of the cellular and molecular mechanism driving neo-alveolarization. Beneficial aspects of this model are represented by the possibility that the advantages of transgenic mice can be used as well. Furthermore, alveolar regrowth can be studied in the adult lung, which might be more directly relevant to lung regeneration of the adult lung. Like studying alveolarization in mice, a disadvantage of studying regrowth after PNX in mice also remains, that driving factors might be different in mice and humans. Furthermore, it has been demonstrated that strongest regrowth takes place in the cardiac lobe [86]. Therefore, it might be necessary to restrict analyses to the cardiac lobe.

### Perspectives

A possible way to approach lung regeneration is to better understand the composition of contributing cell types, the molecular signature and plasticity of cells, and the ECM composition, which together drive alveolarization and neo-alveolarization in order to identify possible candidates for the induction of lung regeneration. Progenitor cells need to be identified. Single-cell genome-wide screening approaches lead to a broader and more complex understanding of cell lineages of different tissue compartments [26, 102]. Better characterization of endodermal and mesenchymal cell lineages including molecular signatures might provide new insights into the cellular hierarchy of alveolarization, and might reveal progenitor cell candidates. Detailed time-dependent lineage tracing and cell depletion approaches covering prenatal and postnatal periods is needed to discover cell type-specific commitment and function during alveolarization. Furthermore, there is need to understand if factors driving bulk alveolarization in the developing lung might also be capable of inducing neo-alveolarization in the adult lung. Cellular and molecular candidates ensuring the homeostasis of the adult lung tissue additionally might be relevant for neo-alveolarization. A further aspect to approach alveolar regeneration might include the impact of aging and senescence on lung homeostasis and repair. As an example, telomerase activity, which impacts on cellular senescence, has been demonstrated to be tissue specifically regulated in mice during development and aging [103]. This is relevant because lung-specific modulation of telomerase activity during lung development might reveal possible target candidates that impact lung regeneration. Furthermore, the identification of possible targets to lung-specifically modulate the process of aging might reveal targets for future therapeutic concepts. Regulation of the cell cycle represents a key mechanism for tissue homeostasis and development. Signal transduction programs of cellular senescence cause an irreversible cell cycle arrest and thus might crucially impact lung regeneration [104]. Apart from processes which drive alveolarization and neo-alveolarization, processes which drive pulmonary diseases have to be considered. Finally, target candidates identified in mice, need to be validated in human tissue.

## Conclusion

**Fig. 4:**
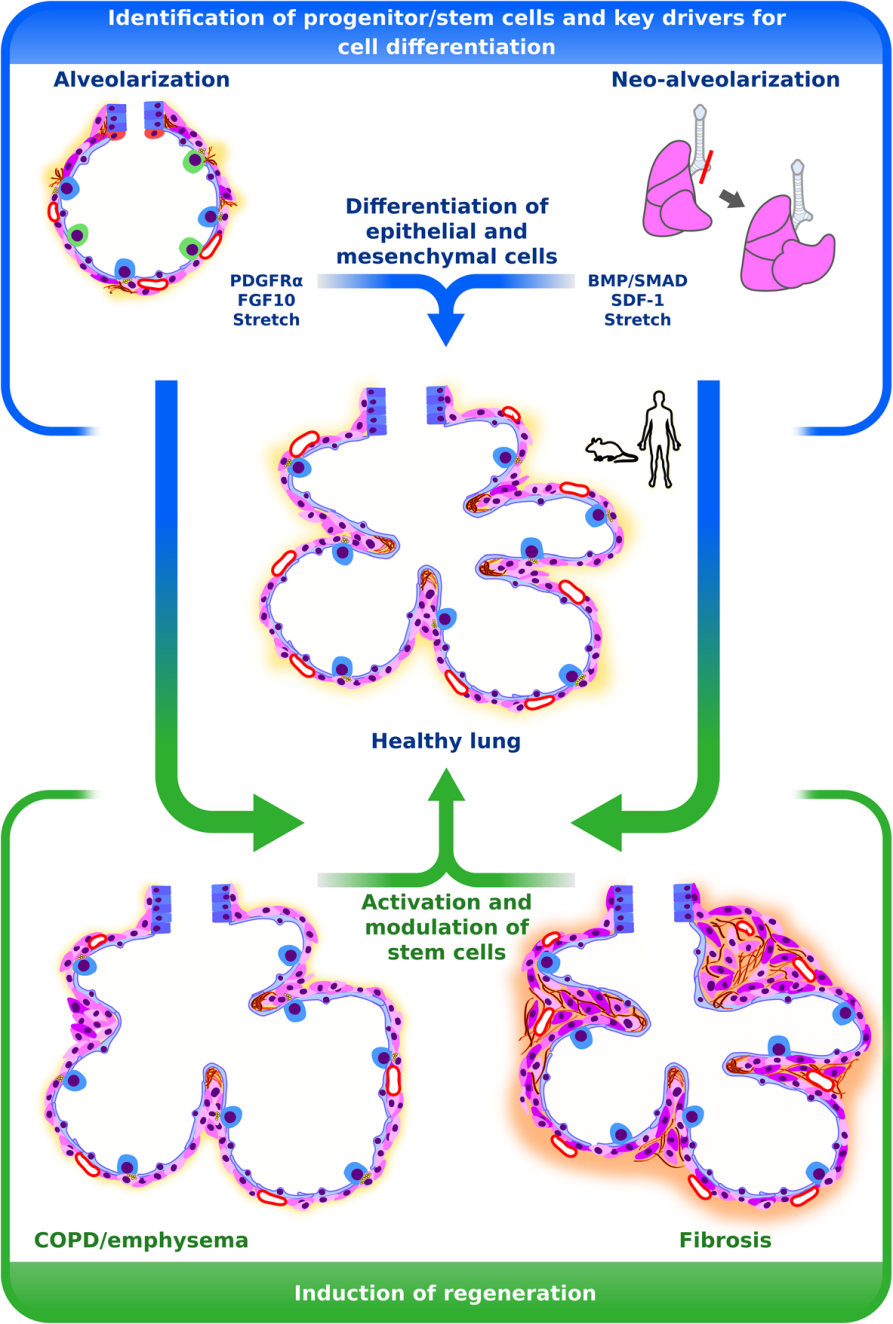
Approaches for lung regeneration. Identification of progenitors or stem cells and mediators driving cellular differentiation during alveolarization and neo-alveolarization might be used to identify possible target candidates to induce regeneration during pulmonary diseases such as emphysema/chronic obstructive lung dsease and fibrosis. Promising target candidates are represented by platelet-derived growth factor receptor (PDGFR)α^+^ cells and fibroblast growth factor (FGF)10 during alveolarization and bone morphogenic protein (BMP))/SMAD family member (SMAD) signaling and stromal cell derived factor (SDF)-1 during neo-alveolarization

Knowledge about alveolarisation and neo-alveolarization will reveal cellular and molecular target candidates that might be exploited for the development of new therapeutic concepts for pulmonary structural diseases. To explore cellular and molecular mediators of alveolarization and neo-alveolarization in vivo*,* suitable tools such as lineage tracing, cell-type specific cell-ablation techniques and cell-type specific molecular modulation, single cell sequencing and high quality 3D imaging approaches are currently available in mice. So far, concerning alveolarization, modulating the PDGFRα^+^ cell-lineage which represents a member of the alveolar stem cell niche and modulating FGF10 which is involved in transducing mechanical forces seem to be promising approaches to modulate alveolarization (Fig. 4). The related candidate of the alveolar niche is represented by AECII. The stem cell function of AECII has been demonstrated to be essential for alveolar regrowth after PNX and has also been highlighted in this review. Identification of factors modulating stem cell functions of AECII and epithelial homeostasis such as BMP/SMAD signaling and SDF-1 represent the most promising approach to understand lung regrowth after PNX and further to develop strategies to induce lung regeneration during pulmonary diseases such as emphysema/COPD and fibrosis (Fig. 4). However, validation of identified candidates and pathways in human material always has to be considered, to ensure the chance of developing new therapeutic concepts for pulmonary diseases in human.

## Abbreviations

3D: Three dimensional
AECI: Alveolar epithelial type I cell
AECII: Alveolar epithelial type II cell
BPD: Bronchopulmonary dysplasia
CD90: Cluster of differentiation 90
COPD: Chronic obstructive lung disease
CRABP: Cellular retinoic acid binding protein
E: Embryonic day
ECM: Extracellular matrix
FGF: Fibroblast growth factor
Gli-1: GLI-Kruppel family member
LGR: Leucine-rich repeat- containing G protein-coupled receptor
P: Postnatal day
PC: Phosphatidyl cholin
PDGF-A: Platelet-derived growth factor-A
PDGF-C: Platelet-derived growth factor-C
PDGFRα: Platelet-derived growth factor receptor-α
PGE2: Prostaglandine E2
PNX: Pneumonectomy
PPARγ: Peroxisome proliferator activated receptor gamma
PTHRP: Parathyriod hormone related peptide
PTHRP: Parathyroid hormone-related peptide
SDF-1: Stromal cell derived factor 1
SMAD: SMAD family member
SPC: Surfactant protein C
TCF21: Transcription factor 21
TGF: Transforming growth factor
Thy-1: Thymus cell antigen 1
αSMA: α−Smooth muscle actin
